# Acute Ophthalmoplegia with Wernicke‐Like MRI Pattern in a Patient with *HPDL* ‐Related Disorder

**DOI:** 10.1002/mdc3.14153

**Published:** 2024-06-28

**Authors:** Jacopo Sartorelli, Daniela Longo, Lorena Travaglini, Valeria Orlando, Adele D'Amico, Enrico Bertini, Francesco Nicita

**Affiliations:** ^1^ Unit of Neuromuscular and Neurodegenerative Diseases, Bambino Gesù Children's Hospital, IRCCS Rome Italy; ^2^ Neuroradiology Unit, Imaging Department Bambino Gesù Children's Hospital, IRCCS Rome Italy; ^3^ Laboratory of Medical Genetics, Translational Cytogenomics Research Unit, Bambino Gesù Children's Hospital, IRCCS Rome Italy

**Keywords:** spasticity, hereditary spastic paraplegia, ataxia, Wernicke encephalopathy, mitochondrial disease, SPG83, thiamine

Biallelic variants in the 4‐hydroxyphenylpyruvate dioxygenase‐like (*HPDL*) gene have been described in 2020 as able to cause a progressive disorder with variable clinical presentation, ranging from severe neonatal‐onset encephalopathy or infantile‐onset neurodegeneration with progressive spasticity and brain white matter abnormalities (NEDSWMA), to milder manifestation of adolescent‐onset pure hereditary spastic paraplegia (HSP) classified as SPG83.^1^

We report the case of a 16.5‐year‐old girl, who firstly came at our attention at 6 months of age for mild lower limb hypertonia and poor social interaction. During the first 3 years of age, she manifested normal motor development with language delay. At around 4 years of age, she developed an ataxic‐spastic syndrome with regression of developmental milestones up to loss of autonomous ambulation at age 5.5 years. Initial brain MRI was unrevealing but progressive cerebro‐cerebellar atrophy appeared and mild bilateral posterior white matter hyperintensity persisted (Fig. 1A–I). Genetic testing performed over years (eg, karyotype analysis, targeted NGS panel for Hereditary Spastic Paraparesis and Spastic Ataxias) was unremarkable. At 14.5 years, she presented acute onset of ptosis, bilateral divergent strabismus and diplopia. Neither infections nor stressful events were reported in the preceding period. During this episode, a further brain MRI showed a Wernicke‐like pattern with bilateral and symmetric FLAIR hyperintensity and T1 hypointensity, without DWI‐ADC restriction and contrast enhancement, of superior colliculi, mesial longitudinal fasciculi, periaqueductal region, interthalamic adhesion and thalamic mesial profile (Fig. 1L–P). Lumbar puncture excluded inflammatory and infectious causes. Multimodal (ie, visual, brainstem auditory and motor) evoked potential revealed an altered conduction of retrochiasmatic optic pathway, upper pons abnormalities and increased central conduction time of pyramidal tracts connected with lower limbs. Metabolic (blood lactate, acylcarnitine, aminoacids, sphingolipids, urinary organic acids, cerebrospinal fluid lactate, aminoacids and organic acids) and vitamin (serum A, B1, B2, B6, B12) screening were all unrevealing. Ex‐adiuvantibus treatment with oral steroids (ie, prednisone 1.3 mg/kg/die, successively tapered in 2 weeks) and antioxidant vitamins (ie, CoQ10, B1, B2 and biotin) was delivered. Resolution of ptosis and diplopia was apparent within 1 week and complete ocular symptoms remission was observed at subsequent visits after 4 months. Brain MRI follow‐up showed improvement of the Wernicke‐like pattern after 2 and 12 months (not shown) and its resolution after 2 years (Fig. 1Q–U). Genetic testing with trio‐whole exome sequencing revealed the homozygous c.365delG (p.Ser122fsTer8) variant in *HPDL* gene (NM_032756.2) inherited from her healthy heterozygous father, classified as likely pathogenetic (class IV) according to ACMG criteria, and not present in current literature nor in variant databases. Successively, SNP array disclosed a long contiguous stretch of homozygosity involving the entire chromosome 1 that was confirmed to have paternal origin indicating paternal isodisomy.

**FIG. 1:**
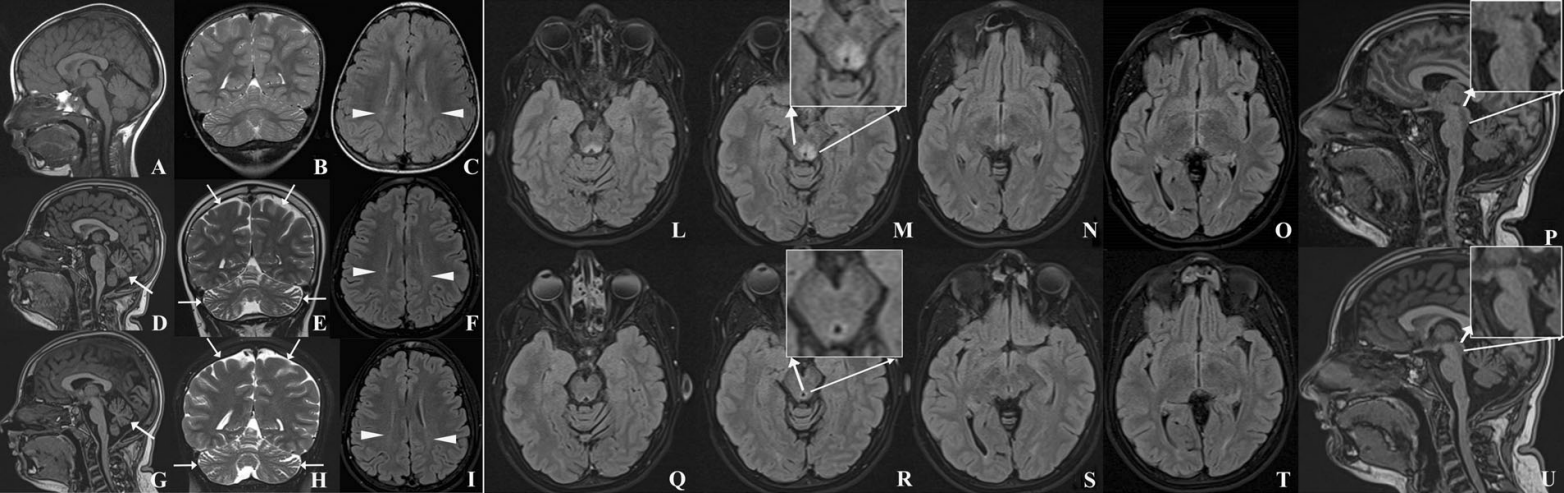
A–I. Brain MRI sagittal T1‐weighted (A, D, G), coronal T2‐weighted (B, E, H) and axial FLAIR (C, F, I) showing progressive cerebro‐cerebellar atrophy (arrows) and mild bilateral posterior white matter hyperintensity (arrowheads) at age 4.5 (A–C), 14.5 (D–F) and 16.5 years (G–I). L–U. Brain MRI axial FLAIR (L–O, Q–T) and sagittal T1‐weighted (P, U) showing Wernicke‐like pattern (magnifications of mesencephalic region) during acute ophthalmoplegia (L–P), with resolution at 2‐year follow‐up (Q–U).

*HPDL*‐related disease has been currently described in 71 subjects from 50 families.^1^, ^2^, ^3^, ^4^, ^5^, ^6^, ^7^, ^8^ Here we have reported a further case with an intermediate form^2^ characterized by early‐onset progressive ataxic‐spastic tetraparesis with neuroimaging feature of progressive brain atrophy with cerebellar predominance, who experienced a sudden episode of ophthalmoplegia with Wernicke‐like pattern during acute phase. Clinical (ie, ophthalmoplegia and ptosis) and neuroimaging (ie, Wernicke‐like pattern) findings resolved over time after steroidal and antioxidant therapy. It is difficult to determine any possible causal link between either of the two treatments and clinical‐radiological improvement, also considering that reversible neuroimaging findings have already been described in *HPDL* patients. In particular, original description suggested three possible MRI patterns^1^: type I with striatal (ie, Leigh‐like) reversible changes with possible lactate peak, and persistent white matter anomalies; type II with predominant relapsing cortical, brain stem, thalamic or inferior colliculi anomalies (similar to the case presented in our study); type III with spinal cord involvement. Pure HSP cases may have normal brain MRI.^2^ Unexplained acute episodes of encephalopathy have also been reported in *HPDL*‐related disorder,^2^ mostly in the severe phenotype, and misdiagnosed with autoimmune disease such as acute disseminated encephalomyelitis.^2^, ^9^ However, clinical features and association with neuroimaging anomalies have not been accurately described yet. Abnormalities in conjugated eye movements have already been reported,^4^, ^10^ but to our knowledge, association between acute ophthalmoplegia and Wernicke‐like neuroimaging pattern is a novel finding.

*HPDL* is an intronless single‐exon gene that encodes the 4‐hydroxyphenylpyruvate dioxygenase‐like protein, which is expressed in mitochondria, and it has been described to play a role in complex II activity and CoQ10 byosynthesis.^10^ Implication of HPDL protein in mitochondrial function and thiamine role in Krebs cycle,^11^ together with similar neuroimaging findings during acute decompensation in both *HPDL*‐related disorder and Wernicke encephalopathy, suggest a possible shared mechanism of energetic dysfunction that still needs to be assessed with novel basic research studies that could clarify *HPDL*‐related disorders pathophysiology.

Finally, broad genetic testing such as exome sequencing is becoming increasingly important in most (neuro‐)genetic disorders including HSPs, due to continuous recognition of novel disease‐causing genes, which may not be detected by non‐updated targeted sequencing. *HPDL* is one of these and it should be considered in both pure and complex forms, where pyramidal signs can be accompanied by ataxia and other transient focal signs up to acute‐onset encephalopathy, and by neuroimaging features of brain atrophy, with hyperintense and possible reversible anomalies of white and deep gray matter, brainstem and spinal cord.

## Author Roles

(1) Research project: A. Conception. B. Data Collection; (2) Manuscript Preparation: A. Writing of the first draft, B. Review and Critique.

J.S.: 2A.

D.L.: 1A, 2B.

L.T.: 1B, 2B.

V.O.: 1B, 2B.

A.D.: 2B.

E.B.: 2B.

F.N.: 1A, 2A.

## Disclosure

**Ethical Compliance Statement:** Submission of the manuscript has been approved by our Institution (approval number RAP‐2023‐0003). Patient's parents have signed the written informed consent for genetic analyses and for participation in study research. We confirm that we have read the Journal's position on issues involved in ethical publication and affirm that this work is consistent with those guidelines.

**Funding Sources and Conflict Of Interest:** This work was supported by the Italian Ministry of Health with “Current Research funds”. The authors declare that there are no conflicts of interest relevant to this work.

**Financial Disclosures for the Previous 12 Months:** The authors declare that there are no additional disclosures to report.

## Data Availability

The data that supports the findings of this study are included in this article.
